# Beyond Genotype: Integrating Phenoconversion into Pharmacogenetic Decision-Making in Psychiatry

**DOI:** 10.3390/ph19101570

**Published:** 2026-10-02

**Authors:** Tiziano Prodi, Maria Gabriela Nielsen, Floriana De Cillis, Nadia Cattane, Moira Marizzoni, Marco Andrea Riva, Bernardo Dell’Osso, Annamaria Cattaneo

**Affiliations:** 1 Department of Pharmacological and Biomolecular Sciences, University of Milan, 20133 Milan, Italy; tiziano.prodi@unimi.it (T.P.);; 2 Department of Psychiatry, ASST Fatebenefratelli-Sacco, 20157 Milan, Italy; 3 Department of Psychiatry, Neomente, 1026 Buenos Aires, Argentina; 4 Department of Mental Health and Psychiatry, Favaloro University, 1093 Buenos Aires, Argentina; 5 Biological Psychiatry Unit, IRCCS Istituto Centro San Giovanni di Dio Fatebenefratelli, 25125 Brescia, Italy; 6 Aldo Ravelli Center for Neurotechnology and Brain Therapeutics, University of Milan, 20142 Milan, Italy; 7 Department of Psychiatry and Behavioral Sciences, Bipolar Disorders Clinic, Stanford University, Stanford, CA 94305, USA; 8 Centro per lo Studio dei Meccanismi Molecolari, Vascolari e Psiconeurobiologici alla Base delle Patologie Neuro-Psico-Geriatriche, University of Milan, 20157 Milan, Italy

**Keywords:** pharmacogenetics, phenoconversion, precision psychiatry, psychopharmacology, cytochrome P450, drug exposure, therapeutic drug monitoring

## Abstract

Interindividual variability in psychotropic drug response contributes to treatment failure, adverse drug reactions, and repeated medication adjustments. Pharmacogenetics supports precision prescribing by identifying genetic variants associated with drug metabolism, efficacy, and tolerability, and evidence-based recommendations are available for several psychotropic medications. However, genotype predicts metabolic capacity rather than the enzyme activity actually expressed in vivo or the systemic drug exposure achieved at a given time. This narrative review examines phenoconversion, the mismatch between genotype-predicted and observed metabolic phenotype, and its implications for psychiatric pharmacotherapy. Drug–drug interactions, smoking, inflammation, physiological changes, and disease-related factors can modify drug-metabolizing enzyme activity or otherwise alter systemic drug exposure despite an unchanged genotype. We discuss how these influences can limit the applicability of genotype-based dosing recommendations and propose a dynamic framework integrating pharmacogenetic results with concomitant medications, clinical conditions, lifestyle factors, therapeutic drug monitoring, and longitudinal changes in patient status. This framework may also refine the assessment of treatment adequacy, because prescribed dose, duration, and adherence do not necessarily ensure adequate drug exposure. Future clinical decision-support systems should combine pharmacogenetic and longitudinal clinical data to enable more individualized and dynamically informed psychopharmacological treatment.

## 1. Introduction

Psychiatric disorders account for a substantial proportion of the global burden of disease and are among the leading causes of disability worldwide [1]. Despite the availability of a broad range of pharmacological treatments, many patients do not achieve a satisfactory response to initial interventions and require multiple treatment adjustments, complicating long-term clinical management [2,3].

Interindividual variability in psychotropic drug response reflects the interplay of multiple biological, clinical, and environmental factors. Genetic variation is one contributor to differences in drug metabolism, therapeutic response, and susceptibility to adverse drug reactions, although its relative contribution varies across medications and clinical outcomes [4]. Pharmacogenetics therefore provides a biologically informed framework for moving beyond a uniform approach to psychopharmacological treatment.

Advances in molecular genetics and clinical pharmacology have enabled the identification of genetic variants affecting drug-metabolizing enzymes, transporters, pharmacological targets, and immune-mediated adverse drug reactions. These discoveries have led to evidence-based prescribing recommendations for an increasing number of gene–drug pairs. In psychiatry, clinically actionable recommendations are available particularly for selected antidepressants and antipsychotics, whereas specific HLA alleles provide clinically relevant information for preventing severe hypersensitivity reactions to selected antiseizure medications used as mood stabilizers [5,6,7].

However, pharmacogenetic testing has an intrinsic limitation: genotype is stable, whereas drug metabolism is dynamic. A genotype-predicted metabolizer phenotype represents the expected metabolic capacity of a pathway in the absence of major modifying influences, but it does not necessarily reflect enzyme activity in vivo when treatment is prescribed or monitored. Concomitant medications, smoking, inflammation, and physiological states such as pregnancy can modify drug-metabolizing enzyme activity. Other factors, including organ dysfunction and changes in body composition, may additionally alter drug distribution or clearance without necessarily changing the metabolic phenotype itself [8,9].

Phenoconversion specifically describes a mismatch between genotype-predicted and observed metabolic phenotype resulting from non-genetic influences [8]. For example, a patient genetically classified as a normal metabolizer may functionally behave as a poor metabolizer during treatment with a potent enzyme inhibitor. Conversely, enzyme induction may increase metabolic activity and reduce exposure to substrates of the affected pathway. Such changes occur without any alteration in the patient’s genotype and may therefore limit the accuracy of prescribing decisions based exclusively on genotype-predicted phenotype.

In this narrative review, we first summarize the pharmacogenetic determinants most relevant to psychopharmacology and then examine phenoconversion as a mechanism through which genotype-predicted metabolic capacity may diverge from currently expressed enzyme activity. We discuss the implications of this dynamic variability for systemic drug exposure, therapeutic drug monitoring (TDM), and pharmacogenetically guided prescribing. Finally, we propose a framework in which pharmacogenetic information is integrated with longitudinal clinical data to support individualized treatment decisions and a more biologically informed assessment of treatment adequacy.

### Literature Search Strategy

Given the narrative scope of this review, a targeted literature search was conducted in PubMed/MEDLINE and in the official online resources of the Clinical Pharmacogenetics Implementation Consortium, the Dutch Pharmacogenetics Working Group, and the U.S. Food and Drug Administration. The final literature search update was conducted on 8 August 2026, before manuscript submission. Search terms included combinations of “pharmacogenetics”, “pharmacogenomics”, “phenoconversion”, “cytochrome P450”, “CYP2D6”, “CYP2C19”, “CYP1A2”, “CYP3A4”, “psychiatry”, “antidepressants”, “antipsychotics”, “mood stabilizers”, “therapeutic drug monitoring”, “drug–drug interactions”, “smoking”, “inflammation”, “obesity”, and “pregnancy”. No initial publication-date restriction was applied, and English-language publications were prioritized. Particular consideration was given to clinical guidelines, systematic reviews, meta-analyses, and studies addressing clinically relevant pharmacokinetic interactions or changes in psychotropic drug exposure. Reference lists of selected publications were also screened to identify additional pertinent studies. Sources were selected according to their relevance to the aims of the review rather than through a formal systematic-review process. Accordingly, no formal record-level screening procedure was conducted, the numbers of retrieved and excluded records were not prospectively recorded, and a PRISMA flow diagram was not considered applicable. No protocol was registered, and no formal risk-of-bias assessment or quantitative evidence synthesis was performed.

## 2. Pharmacogenetic Determinants of Psychotropic Drug Response

Interindividual variability in response to psychotropic medications arises from a complex interplay between genetic and non-genetic factors. Genetic variation may influence pharmacokinetics by affecting drug metabolism and transport, as well as pharmacodynamics by altering drug targets and downstream biological pathways [4].

### 2.1. Pharmacokinetic Genes

Among pharmacokinetic determinants, genetic variation in cytochrome P450 (CYP) enzymes has received the greatest attention and currently represents the principal basis for clinically implemented pharmacogenetics in psychiatry [5,6,9]. CYP2D6 and CYP2C19 are particularly relevant because they contribute to the metabolism of numerous antidepressants and antipsychotics. Depending on the enzyme and the functional consequences of the inherited alleles, individuals may be classified as poor, intermediate, normal, rapid, or ultrarapid metabolizers. These genotype-predicted phenotypes may influence systemic drug exposure and, consequently, the likelihood of therapeutic response or adverse effects.

The clinical relevance of CYP2D6 and CYP2C19 is supported by current Clinical Pharmacogenetics Implementation Consortium (CPIC) recommendations for several serotonin reuptake inhibitor antidepressants. These recommendations also include CYP2B6 for selected medications [5]. Other CYP enzymes contribute substantially to psychotropic drug metabolism but currently have fewer clinically actionable gene–drug associations. CYP1A2, for example, is a major determinant of clozapine and olanzapine metabolism; however, current Dutch Pharmacogenetics Working Group (DPWG) guidelines do not recommend therapy adjustments based on CYP1A2 genotype for either medication. CYP3A4 contributes to the metabolism of several psychotropic drugs, with an actionable DPWG recommendation currently available for quetiapine in CYP3A4 poor metabolizers [6].

Most psychotropic medications are metabolized through more than one enzymatic pathway. Consequently, the pharmacokinetic effect of variation in a single gene depends on the relative contribution of the affected enzyme, the activity of alternative pathways, and the presence of additional factors influencing drug disposition. Pathways beyond CYP-mediated oxidation, including conjugation and drug transport, may also be affected by genetic variation, although their clinical implementation in psychiatric pharmacogenetics remains less established [9]. Therefore, a genotype-predicted phenotype should be regarded as an estimate of metabolic capacity rather than a direct measurement of systemic drug exposure.

### 2.2. Pharmacodynamic and Safety-Related Biomarkers

Genetic variation in pharmacodynamic pathways has also been extensively investigated. Variants in SLC6A4 and HTR2A have been studied in relation to antidepressant efficacy and tolerability. However, current evidence does not support the use of these genes to guide antidepressant prescribing, and CPIC does not provide corresponding clinical recommendations [5]. Associations have also been reported between COMT variants and antipsychotic efficacy or treatment-related adverse effects, but the findings have not resulted in broadly actionable prescribing recommendations [9,10,11].

Similarly, pharmacogenomic studies of lithium response have identified candidate loci, genome-wide associations, and polygenic signatures. Although some findings are promising, their predictive performance and clinical validity remain insufficient to support routine implementation [12].

A clinically important exception is represented by HLA-associated safety biomarkers. Specific HLA alleles are associated with severe cutaneous adverse reactions to selected antiseizure medications, particularly carbamazepine, oxcarbazepine, lamotrigine, and phenytoin [7,13]. In these settings, pharmacogenetic testing is intended primarily to prevent potentially life-threatening hypersensitivity reactions rather than to predict efficacy or optimize systemic drug exposure.

Overall, the current clinical implementation of pharmacogenetics in psychiatry is based predominantly on pharmacokinetic gene–drug associations and selected safety-related biomarkers. This distinction is particularly important because phenoconversion, as defined in this review, concerns the divergence of currently expressed enzyme activity from genotype-predicted metabolic capacity and therefore applies primarily to pharmacokinetic gene–drug associations, rather than to pharmacodynamic markers or HLA-associated immune risk.

## 3. Phenoconversion: When Genotype Does Not Reflect Metabolic Phenotype

Genotype-based inference estimates the metabolic capacity of a drug-metabolizing pathway, whereas metabolic phenotype reflects the enzyme activity actually expressed in vivo. Phenoconversion refers specifically to a mismatch between genotype-predicted and observed metabolic phenotype caused by non-genetic factors [8]. This distinction has important implications for pharmacogenetically guided prescribing. Although an individual’s genotype remains unchanged, metabolic phenotype may vary over time in response to concomitant medications, lifestyle exposures, inflammation, physiological states, and other clinical conditions. If genotype is used as a proxy for current metabolic activity without accounting for these factors, prescribing recommendations may not correspond to the patient’s currently expressed metabolic phenotype.

Importantly, not every non-genetic change in systemic drug exposure constitutes phenoconversion. Changes in absorption, protein binding, distribution, or renal elimination may alter drug concentrations without changing the activity of the genotype-predicted metabolic pathway. Nevertheless, these mechanisms remain clinically relevant because they can further increase the discrepancy between genotype-based expectations and actual drug exposure.

### 3.1. Drug- and Lifestyle-Induced Phenoconversion

Drug–drug interactions are among the most clinically relevant causes of phenoconversion. Concomitant medications may inhibit or induce CYP enzymes, thereby shifting enzyme activity away from that predicted by genotype [14]. Enzyme inhibition may cause a genetically normal metabolizer to function as an intermediate or poor metabolizer, increasing exposure to substrates of the affected pathway.

Fluvoxamine provides a clinically relevant psychiatric example. It inhibits several CYP enzymes, particularly CYP1A2, a major enzyme involved in clozapine metabolism. Concomitant administration may therefore substantially increase clozapine plasma concentrations and the risk of concentration-dependent adverse effects [15,16]. Conversely, enzyme induction may increase enzyme activity and reduce drug exposure. Case-based evidence indicates that carbamazepine administration can markedly reduce clozapine concentrations, probably through the induction of multiple metabolic pathways, potentially compromising treatment efficacy [17].

Phenoconversion may also result from inhibition caused by the substrate drug itself. Paroxetine is both a CYP2D6 substrate and a potent CYP2D6 inhibitor. During repeated administration, progressive CYP2D6 inhibition may reduce paroxetine clearance, contributing to nonlinear pharmacokinetics and time-dependent autoinhibition [15].

CYP activity may also be modified by lifestyle-related exposures. Cigarette smoking is particularly relevant in psychiatry because polycyclic aromatic hydrocarbons produced by tobacco combustion induce CYP1A2 activity. This effect is attributable to combustion products rather than nicotine itself. Smoking can therefore increase the clearance of clozapine and olanzapine and reduce their plasma concentrations [18,19]. Specifically, a meta-analysis estimated that, to achieve equivalent concentrations, olanzapine and clozapine doses would need to be approximately 30% and 50% lower, respectively, in non-smokers than in smokers [18]. Conversely, smoking cessation reverses CYP1A2 induction and may increase drug concentrations, potentially resulting in concentration-dependent toxicity unless the patient is closely monitored and the dose is adjusted when appropriate [20].

### 3.2. Disease-Related Phenoconversion and Other Changes in Drug Exposure

Pathological conditions may alter enzyme activity and contribute to phenoconversion, while also affecting other determinants of drug disposition. Among these conditions, inflammation represents one of the best-characterized mechanisms of disease-related phenoconversion. Proinflammatory cytokines, particularly interleukin-6 and interleukin-1β, can downregulate the expression and activity of several CYP enzymes, including CYP3A4, CYP1A2, CYP2C19, and CYP2D6 [21,22]. Acute or chronic inflammatory states may therefore reduce drug metabolism despite an unchanged genotype.

Inflammation-associated increases in clozapine plasma concentrations have been reported and may increase the risk of concentration-dependent adverse effects [22,23]. Similar mechanisms may affect other substrates of inflammation-sensitive enzymes, including citalopram, risperidone, and quetiapine. However, direct clinical evidence in psychiatry remains limited. Available studies involving clozapine, risperidone, quetiapine, citalopram, and venlafaxine did not adequately characterize pharmacokinetic behavior, and some relied only on dose-normalized serum concentrations or carried a high risk of bias [23]. Consequently, the magnitude and clinical relevance of inflammation-related changes in exposure to psychotropic medications remain uncertain.

Hepatic dysfunction, renal impairment, and critical illness can also alter systemic drug exposure through changes in hepatic blood flow, enzyme or transporter activity, protein binding, and renal clearance [24]. Some of these changes may modify the expressed metabolic phenotype, whereas others affect systemic drug exposure independently of phenoconversion. The distinction is clinically important because genotype-based recommendations cannot account for impaired elimination or altered distribution unless these factors are considered separately.

Obesity provides another example of multifactorial pharmacokinetic variability. Changes in body composition, organ function, and enzyme-specific activity may affect drug distribution and elimination, although the direction and magnitude of these effects are compound-specific [25]. An increased volume of distribution may prolong the elimination half-life of highly lipophilic compounds, but this relationship also depends on drug clearance. Model-based analyses of brexpiprazole suggest that obesity may prolong its half-life and delay attainment of effective concentrations, with potentially greater effects in individuals who are also CYP2D6 poor metabolizers [26]. This example illustrates how genetic and non-genetic determinants may interact, even when the resulting pharmacokinetic change cannot be attributed exclusively to phenoconversion.

### 3.3. Pregnancy as a Dynamic Model of Genotype–Phenotype Interaction

Pregnancy represents a particularly dynamic model of the interaction between genotype-predicted metabolic capacity and non-genetic determinants of drug exposure. Gestational changes affect drug metabolism, distribution, plasma protein binding, and renal elimination, with the magnitude of these effects varying across pregnancy [27,28]. Although the selection of psychotropic medications during pregnancy requires consideration of maternal and fetal safety beyond the scope of this review, pregnancy-related pharmacokinetic changes are highly relevant to the interpretation of pharmacogenetic information and individualized dose optimization.

Pregnancy-associated hormonal changes can induce UGT1A4, the principal enzyme responsible for lamotrigine glucuronidation, leading to increased clearance and reduced lamotrigine concentrations [27]. CYP enzymes are affected in different directions: CYP3A4 and CYP2D6 activities generally increase during pregnancy, whereas CYP1A2 and CYP2C19 activities tend to decrease [27,28]. Consistent with these changes, a TDM study found that dose-adjusted serum concentrations during the third trimester were reduced by 76% for quetiapine and 52% for aripiprazole compared with non-pregnant baseline values [29]. However, these findings reflect the overall effect of pregnancy-related pharmacokinetic changes and cannot be attributed exclusively to changes in a single enzyme.

Pregnancy is also associated with increases in plasma volume, total body water, and adipose tissue, which may alter the volume of distribution. Changes in plasma protein concentrations may additionally modify the unbound fraction of highly protein-bound medications [28]. For example, the unbound fraction of diazepam may increase because of reduced plasma protein binding, meaning that total plasma concentrations may not accurately represent pharmacologically active exposure.

The clinical consequences of these physiological adaptations may depend on the underlying pharmacogenetic profile. Paroxetine provides an illustrative example: pregnancy-related changes in its concentrations have been reported to differ according to CYP2D6 metabolizer status, demonstrating that the same physiological state may produce different pharmacokinetic trajectories across genotype-defined groups [27].

Pregnancy-related changes should also be interpreted in the context of concurrent clinical and lifestyle factors. For example, pregnancy-associated CYP1A2 downregulation and smoking cessation could plausibly have additive effects on exposure to CYP1A2 substrates such as clozapine and olanzapine [20,29]. However, this combined effect has not been systematically quantified and should currently be regarded as a clinically plausible hypothesis rather than an established dosing principle.

Taken together, these observations demonstrate that the clinical interpretation of pharmacogenetic information is inherently dynamic. Although genotype remains constant, metabolic phenotype and systemic drug exposure may change over time in response to environmental exposures, physiological adaptations, and pathological conditions. Pharmacogenetic results should therefore be interpreted alongside the patient’s current clinical status and, when appropriate, TDM.

## 4. From Genotype-Guided Prescribing to Dynamic Clinical Decision-Making

These considerations have important implications for the clinical implementation of pharmacogenetics. International initiatives, particularly the CPIC and the DPWG, have translated pharmacogenetic evidence into prescribing recommendations for several psychotropic medications [5,6,7]. These recommendations provide a foundation for genotype-guided treatment but must be interpreted in the context of the patient’s current pharmacological and clinical status.

### 4.1. Current Pharmacogenetic Prescribing Recommendations

CPIC recommendations are intended primarily to guide medication selection and dosing when pharmacogenetic results are already available. In patients receiving a stable and effective dose without relevant tolerability concerns, treatment changes based solely on a subsequently obtained genotype may not be warranted [5].

For CYP2D6 ultrarapid metabolizers, CPIC recommends selecting an alternative antidepressant not predominantly metabolized by CYP2D6 instead of paroxetine. A similar approach is recommended for vortioxetine; if vortioxetine remains clinically appropriate, treatment can be initiated at the standard starting dose and titrated according to efficacy and tolerability, potentially requiring a higher maintenance dose [5]. By contrast, although CYP2D6 ultrarapid metabolizers show increased conversion of venlafaxine to its active metabolite, O-desmethylvenlafaxine, CPIC does not recommend a genotype-based intervention because evidence of a clinically meaningful effect remains insufficient [5].

For CYP2D6 poor metabolizers, CPIC recommends a 50% reduction in the starting and maintenance doses of paroxetine, together with slower titration. For vortioxetine, treatment should be initiated at 50% of the standard starting dose and titrated to a maximum of 10 mg/day, or an alternative antidepressant not predominantly metabolized by CYP2D6 may be selected. For venlafaxine, CPIC provides an optional recommendation to consider an alternative antidepressant rather than a specific dose reduction, because the clinical consequences of the altered parent drug-to-metabolite ratio remain uncertain [5].

For citalopram and escitalopram, CPIC recommends considering an alternative antidepressant not predominantly metabolized by CYP2C19 in ultrarapid metabolizers. If either medication remains clinically appropriate and response to the standard maintenance dose is inadequate, titration to a higher maintenance dose may be considered. In CYP2C19 poor metabolizers, an alternative antidepressant should be considered; if citalopram or escitalopram is used, CPIC recommends a lower starting dose, slower titration, and a 50% reduction in the standard maintenance dose. For citalopram, the dose should not exceed 20 mg/day in CYP2C19 poor metabolizers because of the risk of QT prolongation [5].

DPWG provides genotype-based recommendations for selected antipsychotics. Dose reductions are recommended for CYP2D6 poor metabolizers receiving aripiprazole, brexpiprazole, haloperidol, pimozide, risperidone, or zuclopenthixol, with additional recommendations for selected intermediate and ultrarapid metabolizer groups. For quetiapine, the actionable association concerns CYP3A4 poor metabolizers rather than CYP2D6 [6]. DPWG does not recommend genotype-guided therapy adjustment for the CYP2D6–clozapine association. In contrast, current U.S. Food and Drug Administration information states that dose reduction may be necessary in CYP2D6 poor metabolizers because clozapine concentrations may be increased [6,30]. Such discrepancies may reflect heterogeneity in the underlying evidence, differences in evidence-assessment frameworks, and the limited availability of prospective clinical data. They highlight the need for further comparative validation and more harmonized recommendations, particularly when non-genetic factors may modify the genotype-predicted phenotype.

For selected antiseizure medications used in psychiatry, pharmacogenetic testing is primarily intended to prevent severe cutaneous adverse reactions rather than to optimize systemic exposure. DPWG considers pre-treatment HLA-B*15:02 testing beneficial when carbamazepine, oxcarbazepine, lamotrigine, or phenytoin is being considered for patients with ancestry from populations in which this allele is sufficiently prevalent. For carbamazepine, testing for HLA-A*31:01 and HLA-B*15:11 may also be considered in patients with relevant ancestry [7]. Positive results generally support selection of an alternative medication, with particularly strong avoidance of carbamazepine in HLA-B*15:02 carriers [7,13].

### 4.2. Integrating Phenoconversion into Prescribing Decisions

Existing pharmacogenetic guidelines necessarily begin with genotype-predicted phenotype. However, CPIC explicitly recognizes that concomitant medications and patient-specific factors may modify the predicted phenotype, and no standardized consensus method has yet been established for adjusting CYP2D6, CYP2C19, or CYP2B6 phenotypes in the presence of inhibitors or inducers [5]. Similar uncertainty applies to inflammation, smoking, pregnancy, and other time-varying determinants of enzyme activity [8,31].

**Figure 1:**
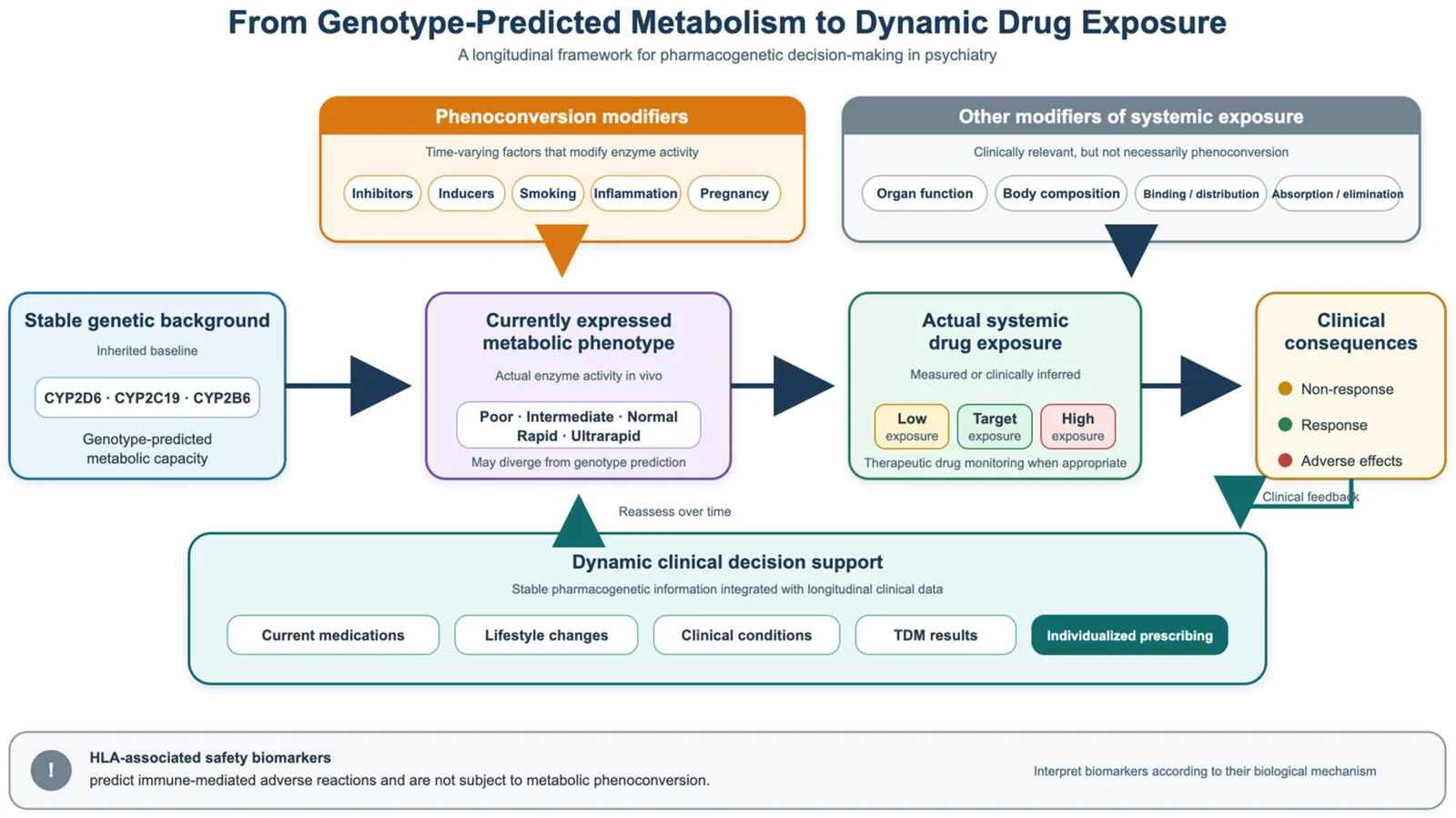
Conceptual framework for precision medicine in psychiatry. Genetic, environmental, lifestyle, and clinical factors shape individual pharmacokinetic and pharmacodynamic variability, contributing to differences in systemic drug exposure, efficacy, and tolerability. The proposed integration of multimodal biological data with longitudinal clinical assessment and therapeutic drug monitoring may support a dynamic precision medicine approach to individualized treatment selection and dose optimization. This figure represents a conceptual framework rather than a validated clinical decision or dosing algorithm.

A clinically useful interpretation should therefore distinguish at least three related levels: genotype-predicted metabolic capacity, currently expressed metabolic phenotype, and measured systemic drug exposure. Genotype informs the first level, clinical and environmental information helps estimate the second, and TDM can assess the third when validated analytical methods and clinically interpretable concentration ranges are available [32]. The relationship between these levels and the longitudinal factors that may modify metabolic phenotype or systemic drug exposure is summarized in Figure 1.

Importantly, for medications with pharmacologically active metabolites, TDM-guided dosing should also consider the active moiety, defined as the combined concentrations of the parent compound and its active metabolite, rather than the parent compound alone, as illustrated by venlafaxine and risperidone. Metabolite-to-parent compound ratios may provide complementary information on in vivo enzyme activity and help identify abnormal metabolism, although their interpretation requires appropriate timing of blood sampling, particularly when the parent compound and metabolite have different elimination half-lives [32].

This approach does not diminish the value of pharmacogenetic testing. Instead, it defines genotype as a stable baseline upon which dynamic factors act. At treatment initiation and during follow-up, clinicians should consider changes in concomitant medications, smoking behavior, inflammatory status, pregnancy, organ function, and other factors that may alter metabolism or drug disposition. Reassessment is particularly relevant when an inhibitor or inducer is started or discontinued, when smoking behavior changes, when an inflammatory illness develops, or when response or tolerability is inconsistent with the genotype-based prediction. Accordingly, until validated adjustment algorithms are available, the identification of a potential modifier should prompt a drug-specific reassessment of systemic exposure, clinical response, and tolerability, supported by TDM when appropriate, rather than automatic phenotype reassignment or dose adjustment.

### 4.3. Toward an Exposure-Informed Concept of Treatment Adequacy

As a hypothesis-generating extension of this framework, the distinction between prescribed dose and systemic drug exposure may have implications for the definition of treatment adequacy. Current definitions of treatment resistance generally operationalize an adequate pharmacological trial according to prescribed dose, treatment duration, and adherence [2,33]. These criteria are clinically useful but do not directly establish whether the individual patient achieved adequate systemic exposure.

A trial may therefore satisfy conventional criteria for dose and duration while producing subtherapeutic exposure because of ultrarapid metabolism, enzyme induction, altered absorption, or other pharmacokinetic factors. Conversely, reduced metabolism or enzyme inhibition may produce excessive exposure and poor tolerability, leading to treatment discontinuation before an effective regimen can be established. In both situations, the prescribed dose alone provides an incomplete representation of pharmacological adequacy.

These considerations support the hypothesis that treatment adequacy could be refined by incorporating evidence of exposure when clinically appropriate. For disorders in which treatment resistance is defined after failure of multiple adequate trials, the relevant question may not be only whether the patient received the recommended dose for a sufficient duration, but also whether that regimen produced adequate exposure for that individual. This exposure-informed concept remains to be prospectively validated and should not currently replace established definitions of treatment resistance. Future studies integrating pharmacogenetic testing, TDM, longitudinal assessment of phenoconversion, adherence, and clinical outcomes will be needed to determine its clinical utility.

## 5. Future Perspectives

The future implementation of pharmacogenetics in psychiatry will require more than the expansion of available gene–drug recommendations. A key priority will be the development of standardized approaches for translating genotype-predicted phenotypes into clinically adjusted phenotypes when inhibitors, inducers, inflammation, smoking, pregnancy, or other modifying factors are present. Although conceptual and quantitative approaches have been proposed, no broadly accepted framework currently integrates these variables across enzymes, medications, and clinical settings [5,31].

Prospective longitudinal studies should assess genotype, concomitant medications, smoking behavior, inflammatory status, organ function, drug concentrations, and clinical outcomes at multiple time points. Such designs would help determine how frequently phenoconversion occurs in psychiatric populations, identify the circumstances in which it becomes clinically relevant, and establish whether changes in metabolic phenotype translate into meaningful differences in efficacy or tolerability. TDM may provide an important link between predicted metabolic capacity and measured systemic drug exposure, particularly for medications with established therapeutic reference ranges [32].

Clinical decision-support systems should ultimately treat pharmacogenetic results as stable baseline information while continuously reassessing dynamic modifiers of metabolism and drug disposition. Importantly, such systems should support a structured evaluation of whether altered drug exposure reflects a true change in metabolic phenotype or other pharmacokinetic or clinical factors. Integration with electronic health records could enable automated review when a relevant medication is initiated or discontinued, smoking behavior changes, an inflammatory illness occurs, pregnancy begins, or hepatic or renal function deteriorates (Figure 1). These systems will require prospective validation to determine whether they improve clinical outcomes, reduce adverse effects, and avoid unnecessary alerts or inappropriate dose adjustments.

Finally, the proposed exposure-informed concept of treatment adequacy should be evaluated in disorder-specific prospective studies. Such research should determine whether the integration of pharmacogenetic data, adherence assessment, TDM, and longitudinal clinical information can distinguish non-response despite adequate drug exposure from treatment failure associated with insufficient exposure. Until these approaches are validated, they should complement rather than replace established clinical definitions of treatment resistance.

## 6. Conclusions

Pharmacogenetics is an increasingly important component of precision psychiatry, with evidence-based prescribing recommendations available for selected antidepressants, antipsychotics, and HLA-associated safety markers [5,6,7,9]. However, genotype-predicted phenotype represents an estimate of metabolic capacity rather than a direct measurement of enzyme activity or systemic drug exposure. Concomitant medications, smoking, inflammation, pregnancy, and other clinical factors may modify the expressed metabolic phenotype, while changes in organ function, protein binding, and body composition may further alter drug exposure independently of phenoconversion.

The limitations introduced by phenoconversion apply primarily to pharmacokinetic gene–drug associations and do not diminish the value of HLA biomarkers used to predict immune-mediated adverse reactions. Pharmacogenetic results should therefore be interpreted according to the biological mechanism of the biomarker and, for metabolic genes, integrated with current medications, lifestyle exposures, physiological conditions, organ function, and TDM when appropriate.

Building on previous work on phenoconversion in psychiatric pharmacogenomics [8,31], this review integrates stable genetic information and longitudinal clinical data within a framework that distinguishes genotype-predicted metabolic capacity, currently expressed metabolic phenotype, and measured systemic drug exposure. Its specific contribution is to apply this distinction to the assessment of treatment adequacy by differentiating the prescribed regimen from the drug exposure actually achieved. Nevertheless, this exposure-informed framework remains a hypothesis requiring prospective validation before it can be incorporated into formal definitions of treatment resistance or routine clinical decision-making.

## Data Availability

No new data were created or analyzed in this study. Data sharing is not applicable to this article.
